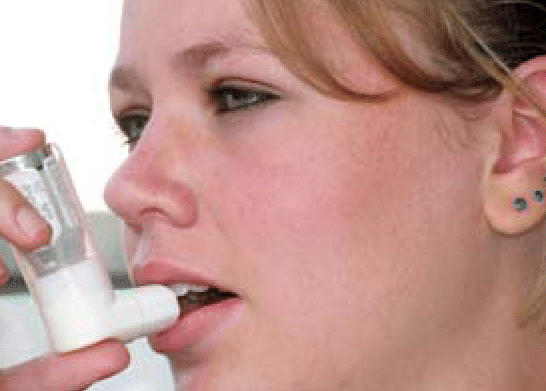# The Beat

**Published:** 2006-04

**Authors:** Erin E. Dooley

## PFOA to Be Eliminated

In January 2006, eight companies agreed to an EPA agreement to eliminate perfluorooctanoic acid (PFOA) from consumer products within the next decade. PFOA, used to make nonstick and stain-resistant materials, has been linked with cancer and birth defects in animals. The chemical has been detected in the blood of 95% of Americans and in marine organisms and polar bears. Currently, PFOA can be found in a wide variety of consumer products, including food packaging, nonstick cookware, and fabrics. Under the terms of the pact, companies will have to reduce manufacturing emissions of PFOA and trace amounts of the compound in consumer products by 95% by no later than 2010. PFOA should be completely eliminated by 2015.

**Figure:**
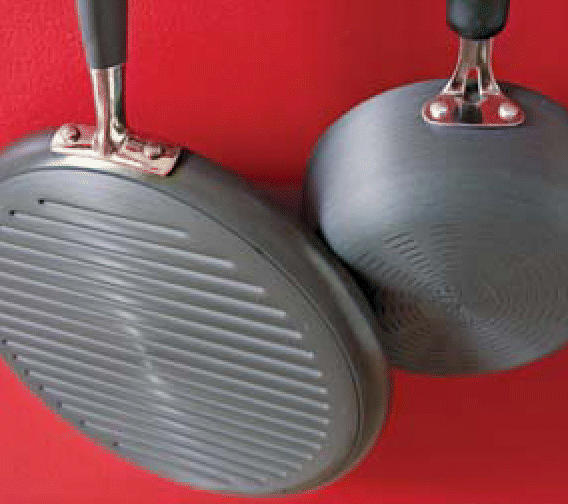


## Mold Genomics

The 22 December 2005 issue of *Nature* featured information on the latest genomes to be cracked: *Aspergillus fumigatus*, the most common infection-causing mold; *A. oryzae*, a nonpathogenic mold that has been used for 2,000 years to make sake, miso, and soy sauce; and *A. nidulans*, widely used as a laboratory model organism. The work to sequence these mold genomes was an international effort, spanning three continents. Scientists working on the project hope their investment will yield insight into the workings of *A. fumigatus*, which could in turn lead to better treatments for serious asthma, allergies, and other conditions in which the fungus is implicated.

## Ahoy There, EPA!

In December 2005, the EPA formally introduced its new Ocean Survey Vessel *Bold*. A converted Navy vessel, the 224-foot ship is a floating scientific laboratory stocked with state-of-the-art equipment to support the EPA’s ocean monitoring and educational tasks, and can accommodate 20 scientists. The EPA began using the *Bold*, its only coastal and ocean monitoring vessel, in August; by September the ship was involved in conducting water quality assessments in the Gulf of Mexico following Hurricane Katrina. The *Bold* will also support enforcement and survey efforts, and function as a nautical classroom, where tours and demonstration events will educate the public about ocean and coastal environmental issues.

**Figure:**
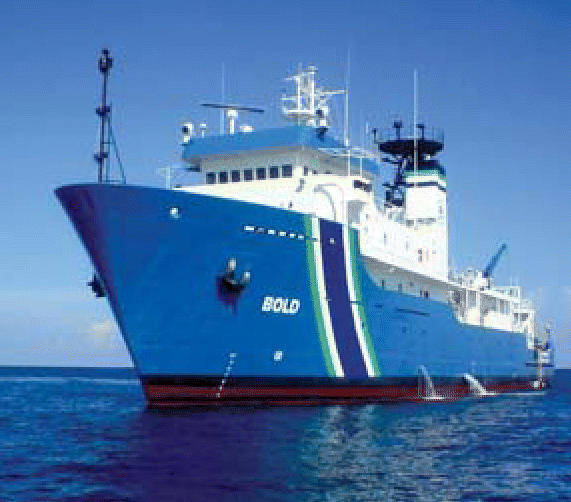


## Zayed Prize Winners

In December the Zayed Prize Higher Committee announced the 2005 winners of this recently established international prize for environmental work. UN Secretary General Kofi Annan was honored for his efforts to catalyze global support for sustainable development. The members of the expert panel of the Millennium Ecosystem Assessment were honored for cataloging the status of the world’s ecosystems and the life-sustaining services they provide. Angela Cropper, co-president of the Cropper Foundation of Trinidad and Tobago, and Emil Salim, the former Indonesian State Minister for Population and Environment, were honored for their efforts to effect actual change in environmental policy. The prizes, worth $1 million apiece, are awarded every two years.

**Figure:**
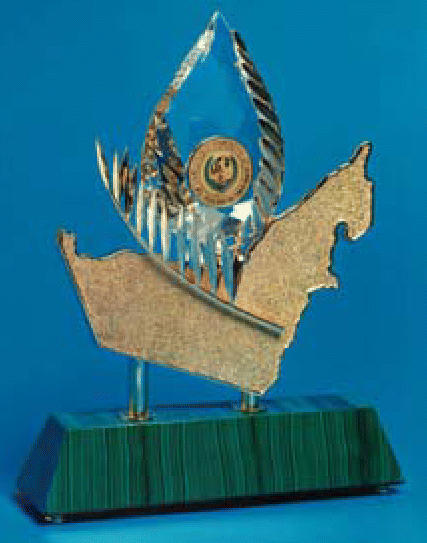


## Testing New Mothers for Toxicants

In fall 2005, the North American Commission for Environmental Cooperation began a continentwide testing program to analyze the blood of 500 first-time mothers for environmental contaminants including dioxins, furans, PCBs, DDT, chlordane, lindane, arsenic, lead, and mercury. The study will give scientists a profile of population exposure to these pollutants and allow them to assess baseline values and potential areas of concern in Mexico. The testing, which is partially funded by the World Bank, will be conducted at 15 sites in Mexico and Canada; pre-existing data will be used for the United States. A report outlining the results of the study is expected in 2006.

## Breathing Easier at School

Now that many state laws allow students to carry asthma and anaphylaxis medications to school and administer these drugs to themselves, the Allergy & Asthma Network Mothers of Asthmatics has launched a campaign to educate students, parents, heath care providers, and school staff about these new laws and to help students better manage their conditions. The campaign homepage at http://www.BreatheAtSchool.org/ offers an interactive U.S. map showing state laws on permitting these medications in schools. Visitors can also download free educational materials such as the Allergies and Asthma at School Kit, which guides students and parents in talking to school staff about allergies and asthma.

**Figure:**